# Supplementary material for: Loss of SUMO-specific protease 2 causes isolated glucocorticoid deficiency by blocking adrenal cortex zonal transdifferentiation in mice
Source: Nat Commun. 2022 Dec 21;13:7858. doi: 10.1038/s41467-022-35526-5 (PMC9772323; doi:10.1038/s41467-022-35526-5)
Supplement: Supplementary file 1 — Supplementary information [file 41467_2022_35526_MOESM1_ESM.pdf]

Supplementary information

Loss of SUMO-specific protease 2 causes isolated glucocorticoid deficiency by blocking adrenal cortex zonal transdifferentiation in mice

Damien Dufour [1], Typhanie Dumontet [1,2,3], Isabelle Sahut-Barnola [1], Aude Carusi [7], Meline Onzon [1], Eric Pussard [4], James Jr Wilmouth [1], Julie Olabe [1], Cecily Lucas [1,5], Adrien Levasseur [1], Christelle Soubeyrand-Damon [1], Jean-Christophe Pointud [1], Florence Roucher-Boulez [1,5], Igor Tauveron [1,6], Guillaume Bossis [7], Edward T.Yeh [8], David T. Breault [9,10], Pierre Val [1], Anne-Marie Lefrançois-Martinez [1] and Antoine Martinez [1*]

[1] institut Génétique, Reproduction & Développement (iGReD), CNRS, INSERM, Université Clermont Auvergne, Clermont–Ferrand, F-63000, France. [2] Department of Internal Medicine, Division of Metabolism, Endocrinology, and Diabetes, University of Michigan, Ann Arbor, Michigan, USA. [3] Training Program in Organogenesis, Center for Cell Plasticity and Organ Design, University of Michigan, Ann Arbor, Michigan, USA. [4] Service de Génétique Moléculaire, Pharmacogénétique et Hormonologie, Hôpital de Bicêtre, Assistance Publique-Hôpitaux de Paris (APHP), Physiologie et Physiopathologie Endocriniennes, INSERM, Université Paris-Saclay, Le Kremlin-Bicêtre, France. [5] Endocrinologie Moléculaire et Maladies Rares, Centre Hospitalier Universitaire, Université Claude Bernard Lyon 1, Bron, France. [6] Service d'Endocrinologie, Centre Hospitalier Universitaire Gabriel Montpied, Université Clermont Auvergne, Clermont-Ferrand, France. [7] IGMM, Université de Montpellier, CNRS, Montpellier, France. [8] Department of Internal Medicine, University of Arkansas for Medical Sciences, Little Rock, Arkansas, USA. [9] Division of Endocrinology, Boston Children's Hospital, Department of Pediatrics, Harvard Medical School, Boston, Massachusetts, USA. [10] Harvard Stem Cell Institute, Harvard University, Cambridge, Massachusetts, USA.

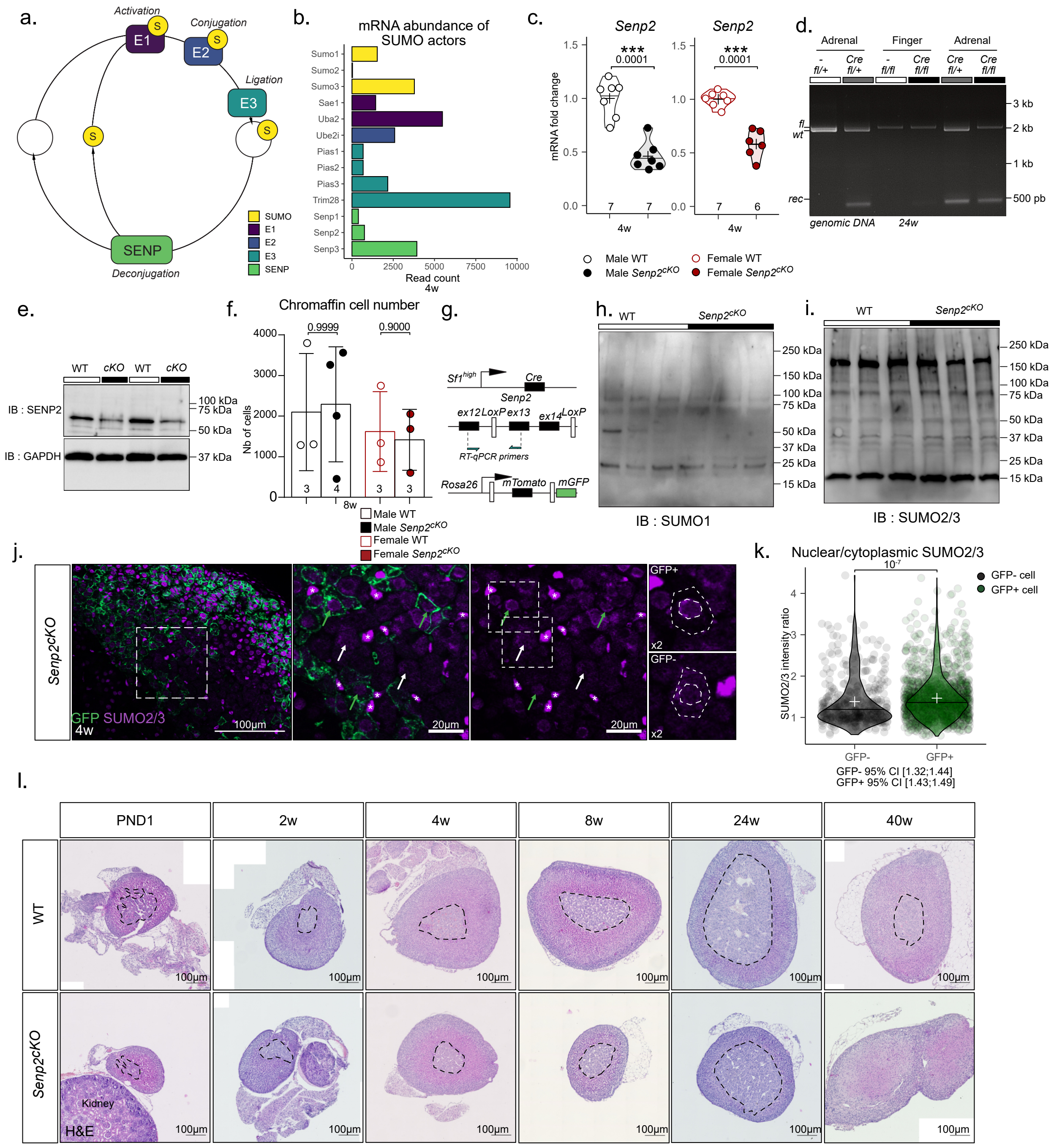

Supplementary figure S 1: **Related to figure 1**

a. Schematic representation of the SUMOylation process

b. Relative abundance of the main actors of SUMOylation in the adrenal gland

c. qPCR analysis of *Senp2* mRNA accumulation in 4-week-old adrenals. P-values were determined by two-sided t.test.

d. Genomic PCR of *Senp2* gene showing specific recombination in *cKO* adrenals

e. Western blot analysis of SENP2 in WT and $Senp2^{cKO}$ 4-week-old male adrenals

f. 2D cell counting number in male and female medulla of WT and $Senp2^{cKO}$ mice. Data are presented as mean values +/- SD.

g. Scheme representing genetic model of $Senp2^{cKO}$ and reporter gene *Rosa26RmTmG*

h. Western blot analysis of SUMO1 in WT and $Senp2^{cKO}$ 4-week-old adrenals

i. Western blot analysis of SUMO2/3 in WT and $Senp2^{cKO}$ 4-week-old adrenals

j. Coimmunofluorescent labelling of GFP (green) and SUMO2/3 (purple) in $Senp2^{cKO}$ adrenal cortex at 4 weeks of age. Asterisks represent endothelial cells

k. Quantification of the ratio of nuclear vs cytoplasmic intensity of SUMO2/3 in GFP-negative cells vs GFP-positive cells in $Senp2^{cKO}$ adrenal cortex

l. Ontogenic analysis of adrenal morphology with H&E staining. Dotted lines represent the boundaries of the medulla.

Source data are provided as a Source Data file * : P-value < 0.05; ** : P-value < 0.01; *** : P-value < 0.001

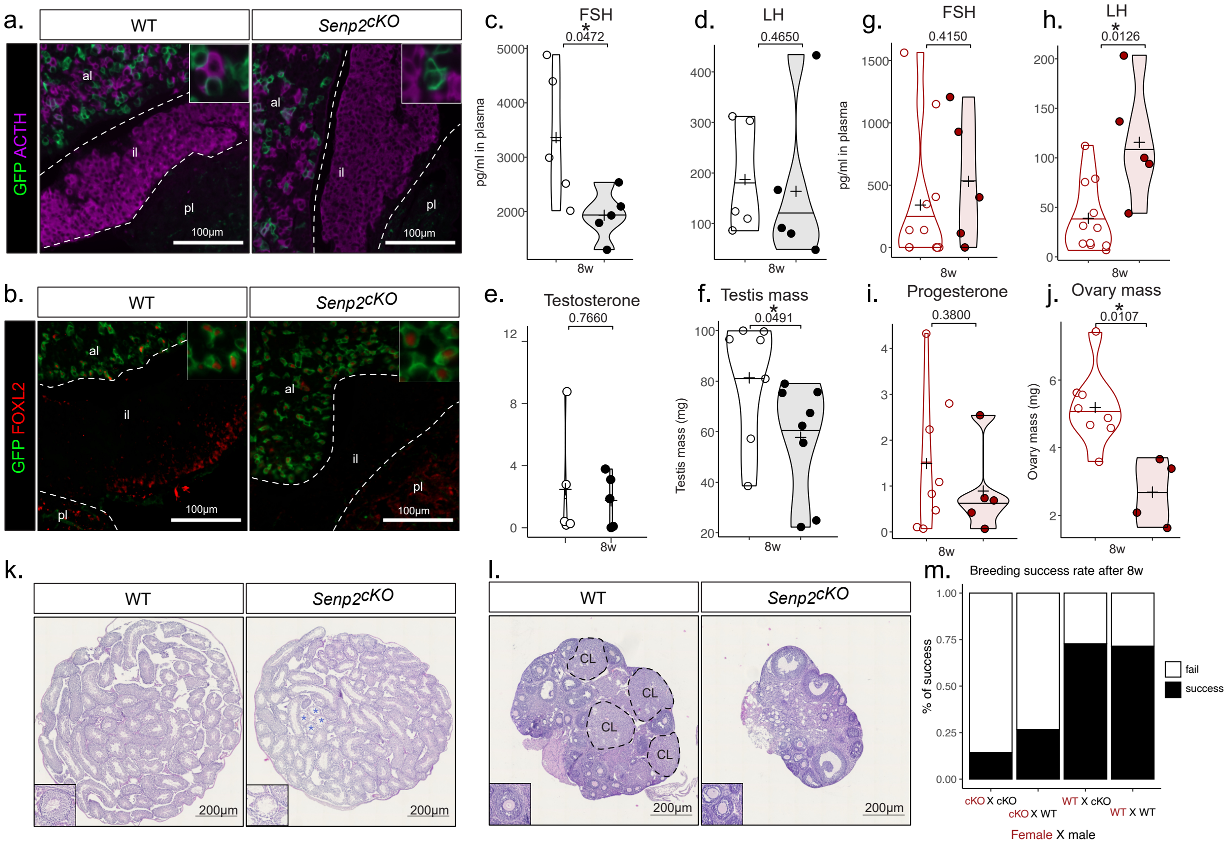

Supplementary figure S 2: **Related to figure 1**
a. Coimmunofluorescent labelling of GFP (green) and ACTH (purple) in WT and *Senp2*^{cKO} 4-week-old pituitaries.
b. Coimmunofluorescent labelling of GFP (green) and FOXL2 (red) in WT and *Senp2*^{cKO} 4-week-old pituitaries.
c-e. Plasmatic levels of FSH (**c.**), LH (**d.**) and testosterone (**e.**) in WT and *Senp2*^{cKO} 4-week-old male mice. *P*-values were determined by two-sided t.test for normally distributed condition or two-sided Mann-Whitney test.
f. Testis mass of WT and *Senp2*^{cKO} 4-week-old male mice. *P*-values were determined by two-sided t.test for normally distributed condition or two-sided Mann-Whitney test.
g-i. Plasmatic levels of FSH (**g.**), LH (**h.**) and testosterone (**i.**) in WT and *Senp2*^{cKO} 4-week-old male mice
j. Ovaries mass of WT and *Senp2*^{cKO} 4-week-old female mice. *P*-values were determined by two-sided t.test for normally distributed condition or two-sided Mann-Whitney test.
k. & **l.** H&E staining of 4-week-old mice's testis (**k.**) and ovaries (**l.**).
m. Reproductive success rate of WT and *Senp2*^{cKO} mice after 8 weeks of breeding.
Source data are provided as a Source Data file * : P-value < 0.05; ** : P-value < 0.01; *** : P-value < 0.001

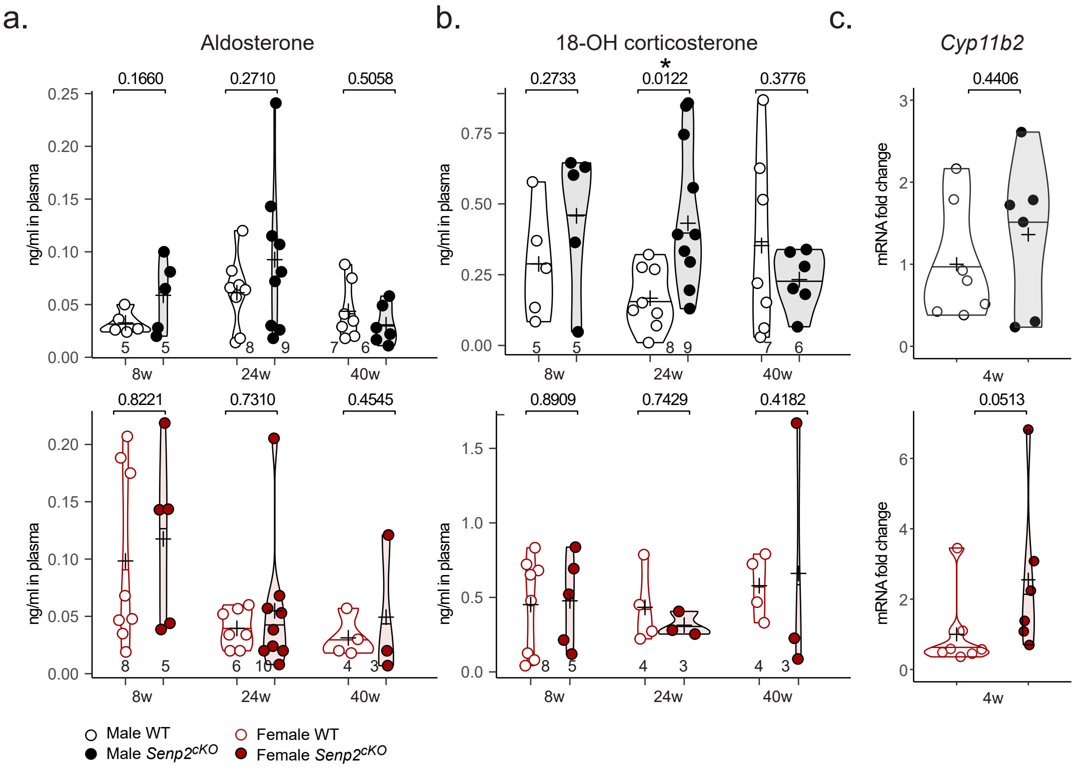

Supplementary figure S 3: **Related to figure 2**

a. Plasmatic concentration of aldosterone in WT and *Senp2*^{cKO} at 8, 24 and 40 weeks of age (determined by LC-MS/MS). P-values were determined by two-sided t.test for normally distributed condition or two-sided Mann-Whitney test.

b. Plasmatic concentration of 18-hydroxy-corticosterone in WT and *Senp2*^{cKO} at 8, 24 and 40 weeks of age (determined by LC-MS/MS). P-values were determined by two-sided t.test for normally distributed condition or two-sided Mann-Whitney test.

c. qPCR analysis of aldosterone synthase coding gene *Cyp11b2* mRNA accumulation in 4-week-old WT and *Senp2*^{cKO} adrenals. P-values were determined by two-sided Mann-Whitney test.

Source data are provided as a Source Data file * : P-value < 0.05; ** : P-value < 0.01; *** : P-value < 0.001

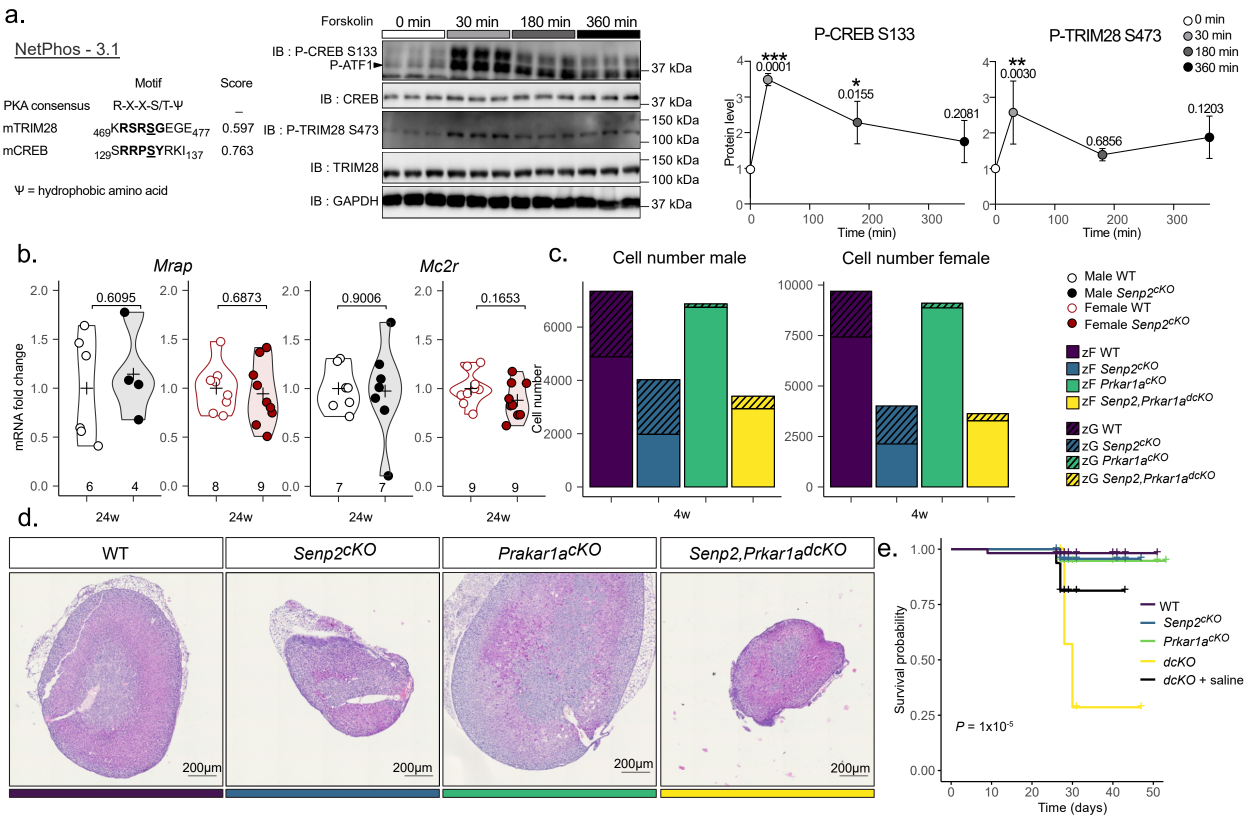

Supplementary figure S 4: **Related to figure 3**

a. Left : PKA phosphorylation motifs on CREB and TRIM28 proteins as determined by NetPhos.3.1.
Right : Western blot analysis of the kinetics of CREB and TRIM28 phosphorylation after forskolin treatment in ATC7 cells. P-values represent difference between wells treated with vehicle compared to 30, 180 and 360 minutes of forskolin. (n=3 wells per condition). Data are presented as mean values +/- SD. Response to forskolin was analysed with two-sided paired 2 way ANOVA to compare the effect of treatment for each condition and adjusted with FDR method.
b. qPCR analysis of ACTH receptor and co-receptor mRNA accumulation in 24-week-old WT and $Senp2^{cKO}$ mice.
c. 2D cell counting number the adrenal cortex of male and female. (n=7 adrenals per condition).
d. Representative H&E staining of WT, $Senp2^{cKO}$, $Prkar1a^{cKO}$ or $Senp2,Prkar1a^{dcKO}$ adrenals
e. Kaplan-Meier curve of double-knockout mice with or without 0.9 % NaCl treatment. P-value was obtained with Log-rank test, χ^2= 28 on 4 degrees of freedom, P = 1x10^{-5}.
Source data are provided as a Source Data file * : P-value < 0.05; ** : P-value < 0.01; *** : P-value < 0.001

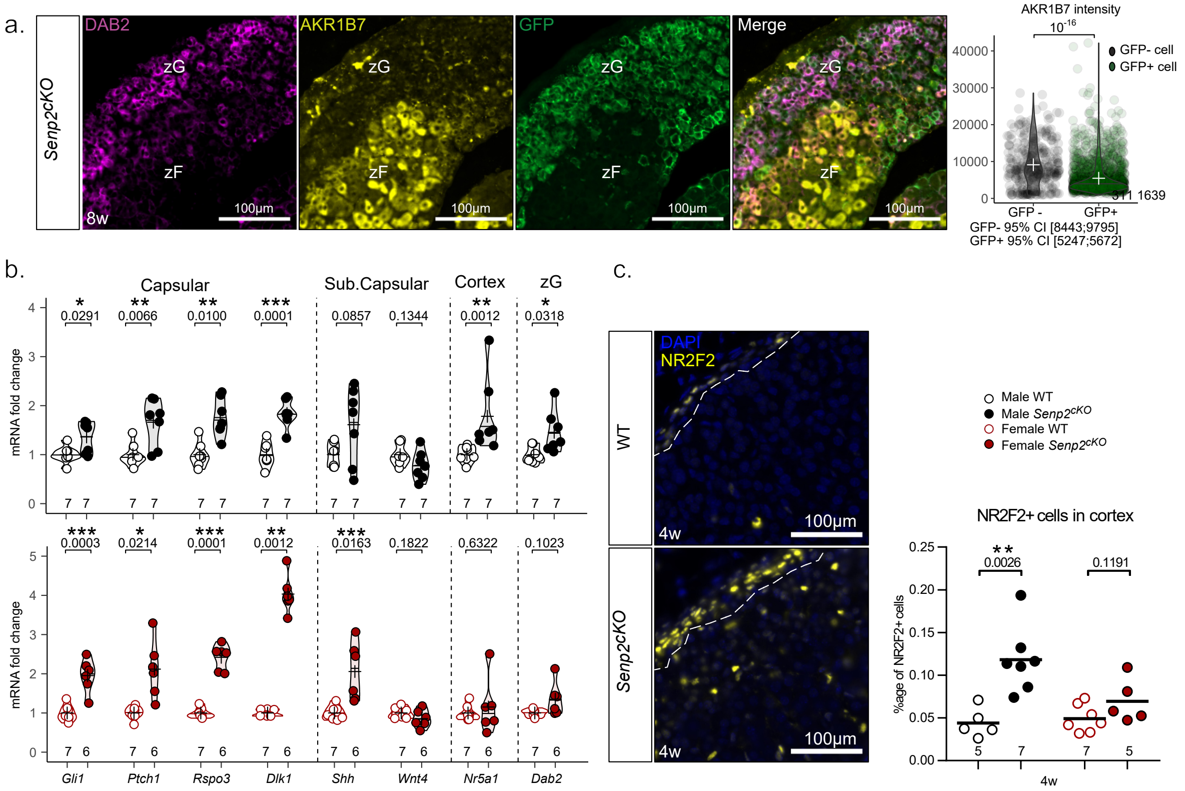

Supplementary figure S 5: **Related to figure 5**

a. Coimmunofluorescent labelling of AKR1B7 (yellow), GFP (green) and Disabled2 (purple) on 8-week-old *Senp2*^{cKO} female adrenal. Quantification of AKR1B7 intensity in GFP+ and GFP- *Senp2*^{cKO} female adrenal cells. *P*-values were determined by two-sided Mann-Whitney test.

b. qPCR analysis of zonal marker mRNA accumulation in 4-week-old WT and *Senp2*^{cKO} adrenals. *P*-values were determined by two-sided t.test for normally distributed condition or two-sided Mann-Whitney test.

c. Immunofluorescent labelling of capsular marker NR2F2 (yellow) with nuclei staining with DAPI (blue) and quantification of the proportion of NR2F2+ cells in the adrenal cortex of WT and *Senp2*^{cKO} male and female adrenal cortices. *P*-values were determined by two-sided Mann-Whitney test.

Source data are provided as a Source Data file * : *P*-value < 0.05; ** : *P*-value < 0.01; *** : *P*-value < 0.001

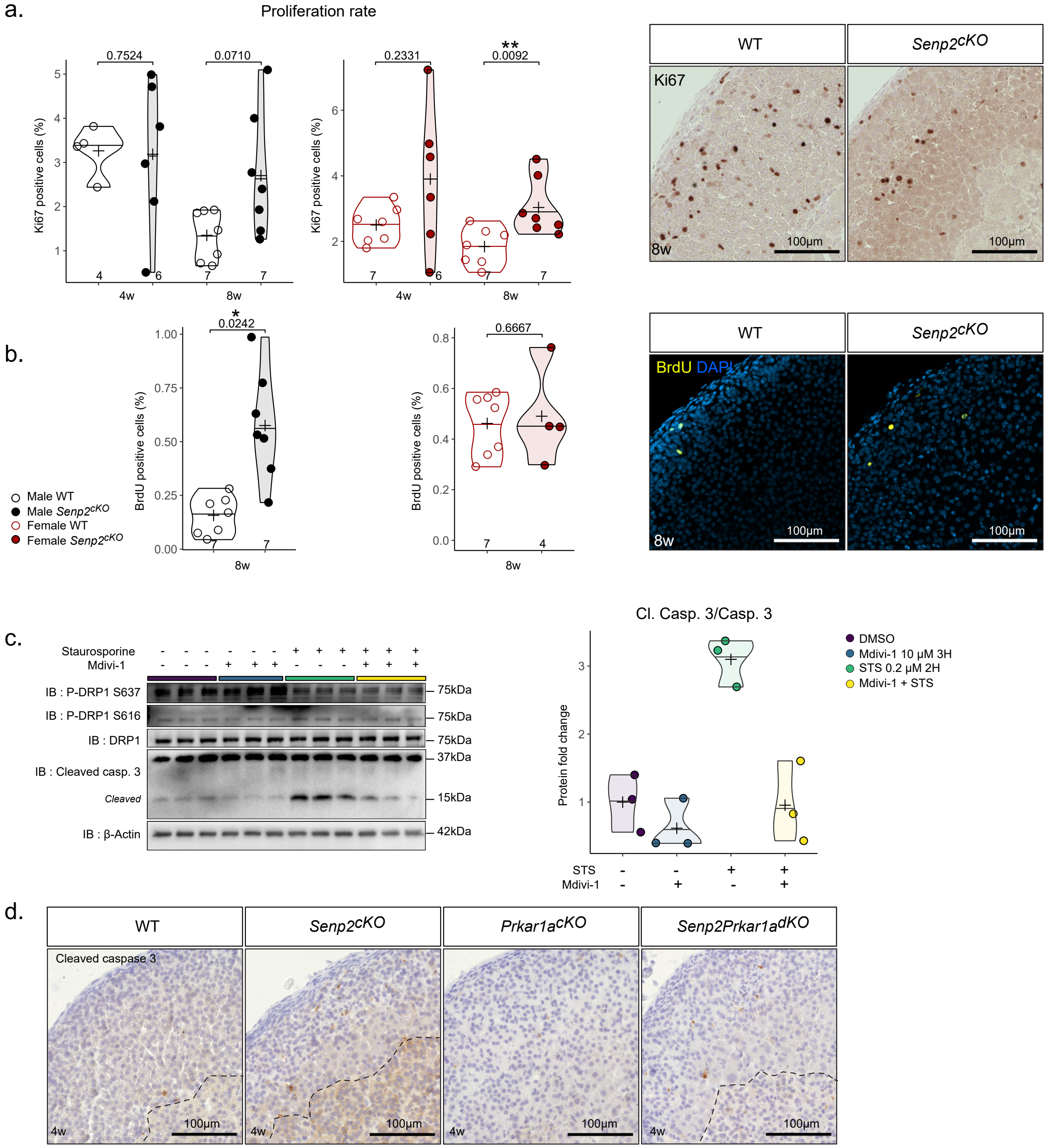

Supplementary figure S 6: **Related to figure 6**

a. Quantification and representative picture of Ki67 staining on WT and *Senp2*^{cKO} 4- and 8-week-old adrenal cortices. *P*-values were determined by two-sided Mann-Whitney test.

b.Quantification and representative picture of 2 hours BrdU incorporation staining on WT and *Senp2*^{cKO} 8-week-old adrenal cortices. *P*-values were determined by two-sided Mann-Whitney test.

c. Western blot analysis of phosphorylated and total DRP1 in cells treated with DMSO, DRP1 inhibitor M-divi1 (10 µM) and/or Staurosporine (0.2 µM). *P*-value was obtained from Kruskal-Wallis test and adjusted with FDR method.

d. Immunostaining of cleaved caspase3 staining of WT, *Senp2*^{cKO}, *Prkar1a*^{cKO} or *Senp2,Prkar1a*^{dcKO} adrenals

Source data are provided as a Source Data file * : *P*-value < 0.05; ** : *P*-value < 0.01; *** : *P*-value < 0.001

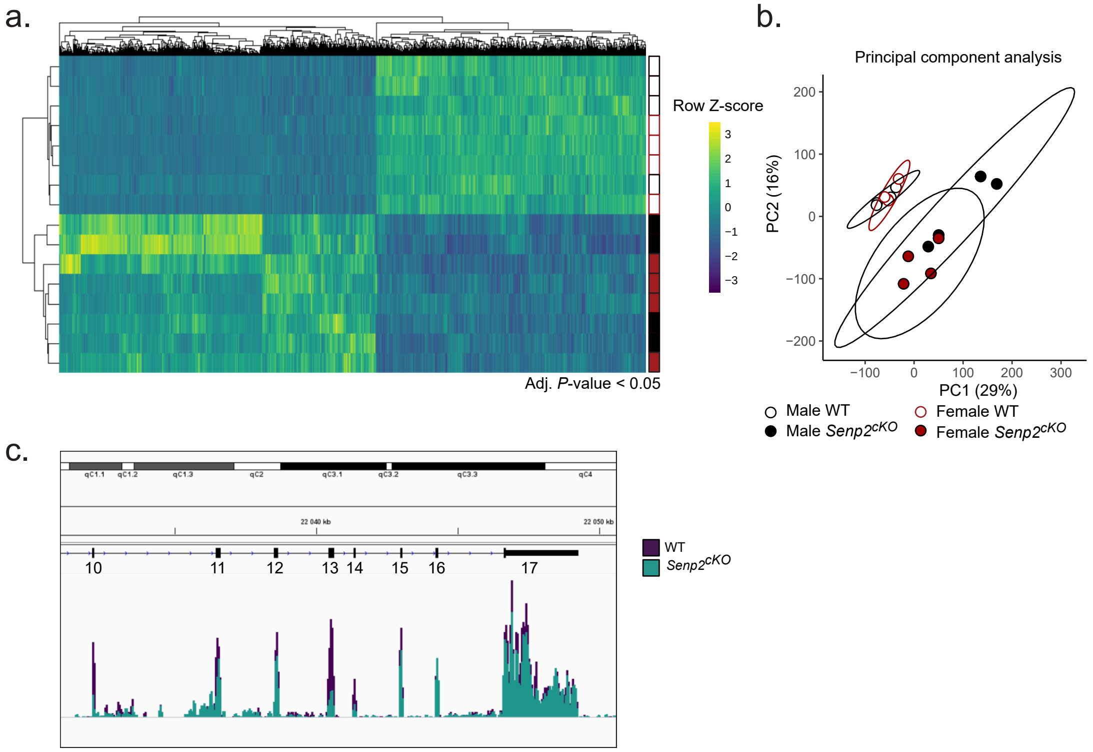

Supplementary figure S 7: **Related to figure 7**

a. Heatmap representing the median centred expression of dysregulated genes (adjusted *P*-value < 0.05 determined with Benjamini and Hochberg method, between WT and *Senp2*^{cKO} male or female) in 4-week-old male and female, WT and *Senp2*^{cKO} adrenals

b. Principal component analysis of gene expression in WT and *Senp2*^{cKO} 4-week-old male and female adrenals

c. Genomic alignment of RNA reads from WT and *Senp2*^{cKO} samples

Source data are provided as a Source Data file

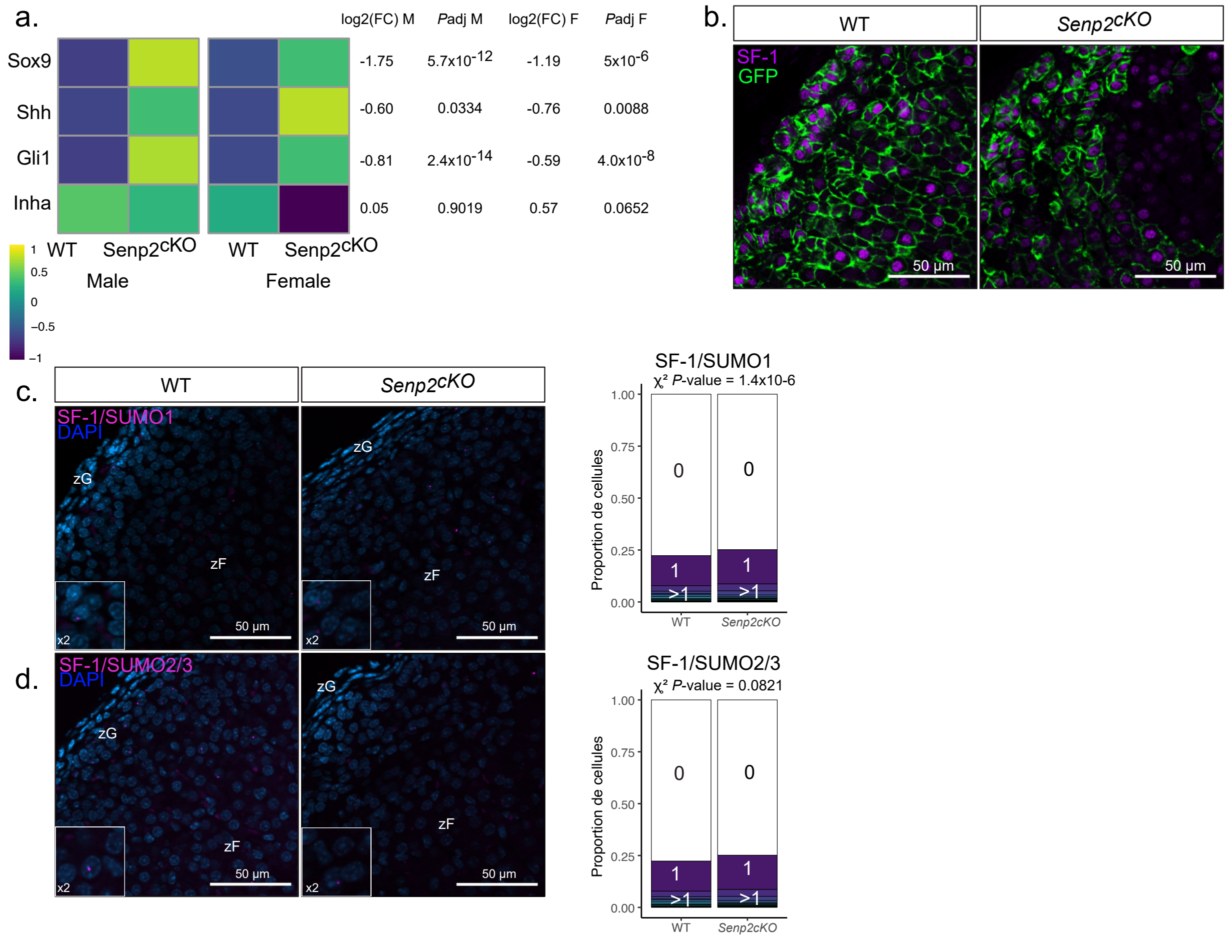

Supplementary figure S 8: **Related to discussion**

a. Heatmap showing expression of "SUMO-sensitive" genes in WT and *Senp2*^{cKO} 4-week-old male and female adrenals. Adjusted P-values were determined with Benjamini and Hochberg method.

b. Coimmunofluorescent labelling of SF-1 (purple) and GFP (green) in WT and *Senp2*^{cKO} 4-week-old adrenals

c. & **d.** Proximity ligation assay showing low SUMO1- (**c.**) and SUMO2/3-ylation (**d.**) of SF-1 in WT and *Senp2*^{cKO} 4-week-old adrenals.

Source data are provided as a Source Data file

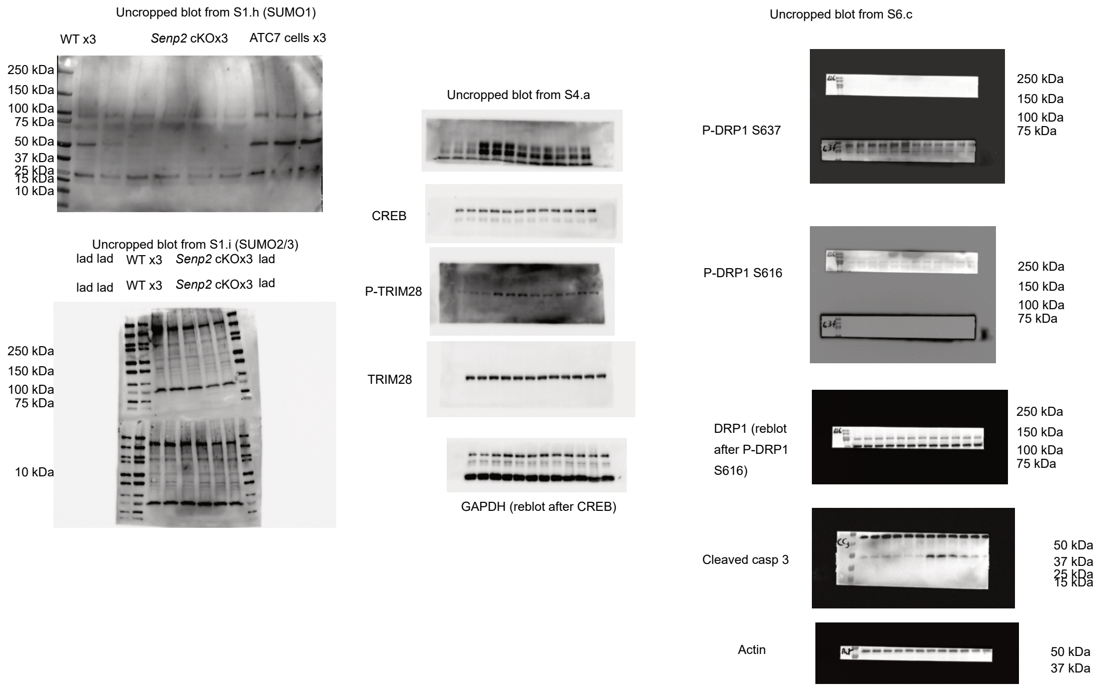

Supplementary figure S 9: **Uncropped gels from supplementary figures**

Gene name	Fw	Rv
Akr1b7	GCCAGTGACCAACCAGATTGAGA	ACGGGGTCTTCTGGCTTGGCAT
Apcdd1	CTCAGCCCCACACTCATTCC	TGGCACGGAGTTTGTGTTCA
Axin2	TGGGGAGTAAGAAACAGCTCC	AGCCTTTGACCAGCACTGAG
Ccdc80	AGGCATGCAATTTTGGTCTGC	ACATCTTCCCGCTCAACGAT
Cyp11a1	CTGCCTCCAGACTTCTTTCG	TTCTTGAAGGGCAGCTTGTT
Cyp11b1	GCAGAGATGATGCTCCTGCTT	GAGAGGGCAATGTGTCATCAGAA
Cyp11b2	ATGCTGAGAAGTTGCACCAG	ATTCTGGCCCATTTAGCAAG
Cyp21a1	GCTGTGGCTTTCCTGCTTCAC	GGCCCAGCTTGAGGTCTAACT
Dab2	CCTGCATCTTCTGATCCCCAC	CATGTTTCTGGCTGTCTGCTT
Dlk1	CTCCTGCGCGTCCTCTTGCTC	CTGCAGACATTGTCAGCCTCGCAG
Gli1	CCTGGTGGCTTTCATCAACTCTCG	CACAGGGCTGGACTCCATAGG
Hsd3b1	ATGGTCTGCCTGGGAATGAC	ACTGCAGGAGGTCAGAGCT
Lef1	GACGAGCACTTTTCTCCGGG	TGGGGTGATCTGTCCAACGC
Mc2r	CAAACACCACCCCCGTCTTA	TCTTGCGGTGTCATTGGTGT
Mrap	CAGAAGCCCTACAGGGGAAC	AGAATCACCCGGCTTGTCTG
Nr5a1	TGCAGAATGGCCGACCAG	TGGCGGTAGATGTGGTC
Ptch1	CCATACACCAGCCACAGCTTCG	GGAGGCTGGAGTCTGAGAACTG
Rspo3	TCATTTTGAACTTTATGGAATACATTG	CAGCCATTGTAATCTGAACACG
Scarb1	CCTTCGTGGAGAACCGCAGCC	CCCATGGTGACCAGCGCCAA
Senp2	GCGGAGACATCCAGACCTTA	AGGCTCCAATGTACCTTCCG
Shh	GCGGCAGATATGAAGGGAAGATC	GTTCATCACAGAGATGGCCAAGGC
Star	TCGCTACGTTCAAGCTGTGT	ACGTCGAACTTGACCCATCC
Wnt4	CCCTGTCTTTGGGAAGGTGGTG	CACCTGCTGAAGAGATGGCGTATAC
36b4	GTCACTGTGCCAGCTCAGAA	CAATGGTGCCTCTGGAGAT
Senp2 (genomic DNA)	ACTTCACAACAGTGAGGACT	AAGTGCAGGAGGAGGTGGATTCAA
Senp2 (genomic DNA)	CTTCTGCTTCTCTTAGTGCT	AAGAGCAAGCACTCTTACTG

Supplementary table S 1: **List of primers used in the study**

IHC

Antibody	Manufacturer	Reference	host	unmasking	Saturation	dilution
AKR1B7	Santa Cruz	SC-27763	goat	CT	Horse serum 2.5%	1/200
TH	Antibodies online	AA 30 100	goat	CT	Horse serum 2.5%	1/1000
TH	Chemicon	AB 152	rabbit	CT	Horse serum 2.5%	1/500
DAB2	BD bioscience	610464	mouse	CT	Horse serum 2.5%	1/500
Laminin	Sigma Aldrich	L9393	rabbit	TE	Horse serum 2.5%	1/200
GFP	invitrogen	A11122	rabbit	CT	Horse serum 2.5%	1/1500
GFP	Abcam	Ab5450	goat	CT/TE	Horse serum 2.5%	1/1000
Tomato/RFP	Rockland	600-401-379	rabbit	TE	Horse serum 2.5%	1/1000
SF1	Cosmo Bio	KAL-KO610	rat	TE	Horse serum 2.5%	1/100
CYP21	Sigma Aldrich	HPA 048979	rabbit	CT	Horse serum 2.5%	1/500
Cleaved Caspase3	Cell signaling	CST9661	rabbit	CT	Horse serum 2.5%	1/200
Active β-catenin	Cell signaling	CST4270	rabbit	CT	Horse serum 2.5%	1/500
β-catenin	BD bioscience	397555	mouse	CT	Horse serum 2.5%	1/500
SUMO2/3		8A2	mouse	CT	Horse serum 2.5%	1/200
NR2F2	Perseus	PPDH7147D00!	mouse	V	Horse serum 2.5%	1/200
Ki67	Abcam	Ab15580	rabbit	CT	Horse serum 2.5%	1/3000
BrdU	Roche	11170376001	mouse	CT	Horse serum 2.5%	1/400
SUMO2/3	Abcam	Ab3742	rabbit	CT	Horse serum 2.5%	1/200

PLA

Antibody	Manufacturer	Reference	host	unmasking	Saturation	dilution
PKA Cαβ	BD bioscience	610981	mouse	CT	PLA kit blocking solution	1/100
GATA6	Cell signaling	CST5851	rabbit	CT	PLA kit blocking solution	1/200
SF1	Cosmo Bio	KAL-KO611	rabbit	CT	PLA kit blocking solution	1/50
SUMO2/3	Abcam	Ab3742	rabbit	CT	PLA kit blocking solution	1/100
β-catenin	BD bioscience	397555	mouse	CT	PLA kit blocking solution	1/200
SUMO2/3		8A2	mouse	CT	PLA kit blocking solution	1/100

Western Blot

Antibody	Manufacturer	Reference	host	Saturation	dilution
P-CREB S133	Cell signaling	CST9198	rabbit	BSA 5%	1/500
CREB	Cell signaling	CST9197	rabbit	BSA 5%	1/1000
β-actin	Sigma-Aldrich	2066	rabbit	BSA 5%	1/10000
P-DRP1 S637	Cell signaling	CST4867	rabbit	BSA 5%	1/200
P-DRP1 S616	Cell signaling	CST3455	rabbit	BSA 5%	1/200
DRP1	NOVUS	NB55237	rabbit	BSA 5%	1/500
Cleaved Caspase3	Cell signaling	CST9661	rabbit	BSA 5%	1/500
β-catenin	BD bioscience	397555	mouse	BSA 5%	1/1000
SUMO2/3		8A2	mouse	BSA 5%	1/200
SUMO2/3	Abcam	Ab3742	rabbit	BSA 5%	1/500
GAPDH	NOVUS	NB30021	rabbit	BSA 5%	1/10000
SUMO1	Cell signaling	CST4930	rabbit	BSA 5%	1/500
TRIM28	Bethyl	BETA700-014-T	rabbit	BSA 5%	1/1000
P-TRIM28 S473	Biolegend	BLE654101	mouse	BSA 5%	1/1000
PKA Cαβ	BD bioscience	610981	mouse	BSA 5%	1/500
HA	Abcam	ab9110	rabbit	BSA 5%	1/5000

CT = sodium citrate 10mM tween 0.01% pH=6 antigen retrieval
TE = Tris 10mM EDTA 1mM pH= 9 antigen retrieval
V = Vector unmasking solution (H330, Vector Labs)

Supplementary table S 2: **List of antibodies used in the study**
