# Supplementary material for: Loss of SUMO-specific protease 2 causes isolated glucocorticoid deficiency by blocking adrenal cortex zonal transdifferentiation in mice
Source: Nat Commun. 2022 Dec 21;13:7858. doi: 10.1038/s41467-022-35526-5 (PMC9772323; doi:10.1038/s41467-022-35526-5)
Supplement: Supplementary file 3 — Reporting Summary [file 41467_2022_35526_MOESM3_ESM.pdf]

nature portfolio

Corresponding author(s): Antoine Martinez

Last updated by author(s): Nov 21, 2022

Reporting Summary

Nature Portfolio wishes to improve the reproducibility of the work that we publish. This form provides structure for consistency and transparency in reporting. For further information on Nature Portfolio policies, see our Editorial Policies and the Editorial Policy Checklist.

Statistics

For all statistical analyses, confirm that the following items are present in the figure legend, table legend, main text, or Methods section.

n/a	Confirmed	
☐	☒	The exact sample size (*n*) for each experimental group/condition, given as a discrete number and unit of measurement
☐	☒	A statement on whether measurements were taken from distinct samples or whether the same sample was measured repeatedly
☐	☒	The statistical test(s) used AND whether they are one- or two-sided<br>*Only common tests should be described solely by name; describe more complex techniques in the Methods section.*
☐	☒	A description of all covariates tested
☐	☒	A description of any assumptions or corrections, such as tests of normality and adjustment for multiple comparisons
☐	☒	A full description of the statistical parameters including central tendency (e.g. means) or other basic estimates (e.g. regression coefficient) AND variation (e.g. standard deviation) or associated estimates of uncertainty (e.g. confidence intervals)
☐	☒	For null hypothesis testing, the test statistic (e.g. *F*, *t*, *r*) with confidence intervals, effect sizes, degrees of freedom and *P* value noted<br>*Give P values as exact values whenever suitable.*
☒	☐	For Bayesian analysis, information on the choice of priors and Markov chain Monte Carlo settings
☒	☐	For hierarchical and complex designs, identification of the appropriate level for tests and full reporting of outcomes
☒	☐	Estimates of effect sizes (e.g. Cohen's *d*, Pearson's *r*), indicating how they were calculated

Our web collection on statistics for biologists contains articles on many of the points above.

Software and code

Policy information about availability of computer code

Data collection	data collection was done by using ZEN 3.4 blue edition software and QuPath 0.3.2
Data analysis	Software used for data analysis were R 4.2.1 and g:profiler. Image analysis and base calling were performed using RTA 2.7.3 and bcl2fastq 2.17.1.14. Adapter dimer reads were removed using DimerRemover. Reads were mapped onto the mm10 assembly of Mus musculus genome using STAR version 2.5.3a. Read counts have been normalised across samples with the median-of-ratios method proposed by Anders and Huber. Gene expression quantification was performed from uniquely aligned reads using htseq-count version 0.6.1p1. Differential expression have been implemented using the Bioconductor package DESeq2 version 1.16.1. Raw and processed data have been deposited in NCBI's GEO database (GSE193480). The code used to generate figure 4.c. 7.a. and supplementary figure 7.b., 8.c. and 8.d. are available on GitHub Doi : 10.5281/zenodo.7347553. Packages used: Bioconductor, sumo, stats, Rtoolbox, Pheatmap, Vennerable, ggplot2.

For manuscripts utilizing custom algorithms or software that are central to the research but not yet described in published literature, software must be made available to editors and reviewers. We strongly encourage code deposition in a community repository (e.g. GitHub). See the Nature Portfolio guidelines for submitting code & software for further information.

Data

Policy information about availability of data

All manuscripts must include a data availability statement. This statement should provide the following information, where applicable:

- Accession codes, unique identifiers, or web links for publicly available datasets
- A description of any restrictions on data availability
- For clinical datasets or third party data, please ensure that the statement adheres to our policy

Sequencing reads were mapped onto the mm10 assembly of Mus musculus genome. Sequencing data has been deposited in GEO with the accession code GSE193480. All other data that supports the findings of this study is provided in the article or supplementary data. Source data is provided with this paper.

Human research participants

Policy information about studies involving human research participants and Sex and Gender in Research.

Reporting on sex and gender	n/a
Population characteristics	n/a
Recruitment	n/a
Ethics oversight	n/a

Note that full information on the approval of the study protocol must also be provided in the manuscript.

Field-specific reporting

Please select the one below that is the best fit for your research. If you are not sure, read the appropriate sections before making your selection.

☒ Life sciences ☐ Behavioural & social sciences ☐ Ecological, evolutionary & environmental sciences

For a reference copy of the document with all sections, see nature.com/documents/nr-reporting-summary-flat.pdf

Life sciences study design

All studies must disclose on these points even when the disclosure is negative.

Sample size	Sample size were determined according to our preliminary data
Data exclusions	No data was excluded
Replication	All experiments have been repeated at least twice of thrice with consistent results. Immunostaining pictures are representative of a group of at least 5 replicates.
Randomization	Cohorts of experimental animals for phamacological studies using SUMO E1 inhibitor (TAK-981) or ACTH were randomised. For all other experiments, experimental allocation was determined by genotype and wild-type littermates were used as control to blunt any confounding variable.
Blinding	Treatments were done without knowing the genotype.

Reporting for specific materials, systems and methods

We require information from authors about some types of materials, experimental systems and methods used in many studies. Here, indicate whether each material, system or method listed is relevant to your study. If you are not sure if a list item applies to your research, read the appropriate section before selecting a response.

Materials & experimental systems

n/a	Involved in the study
☐	☒ Antibodies
☐	☒ Eukaryotic cell lines
☒	☐ Palaeontology and archaeology
☐	☒ Animals and other organisms
☒	☐ Clinical data
☒	☐ Dual use research of concern

Methods

n/a	Involved in the study
☒	☐ ChIP-seq
☒	☐ Flow cytometry
☒	☐ MRI-based neuroimaging

Antibodies

Antibodies used

Condition used are detailed in Supplementary Table S2

Validation

Active β-catenin (Cell signaling CST4270) Non-phospho (Active) β-Catenin (Ser33/37/Thr41) Antibody recognizes endogenous β-Catenin when residues Ser33, Ser37, and Thr41 are not phosphorylated. It does not detect β-Catenin if tri-phosphorylated at Ser33/Ser37/Thr41. This antibody may also detect β-Catenin protein when singly phosphorylated at Ser33. This specificity data was derived from competition ELISA and dot blot analysis using synthetic peptides. (cited 81 times in the litterature)
AKR1B7 (Santa Cruz SC-27763) (cited 9 times in the litterature)
β-actin (Sigma-Aldrich 2066) Anti-Actin specifically stains typical stress fibers in cultured chicken fibroblasts. (cited 3379 times in the litterature)
β-catenin (BD bioscience 397555) routinely tested in western blot, tested during development in IHC, immunoflorescence and IP by BD bioscience. (cited 6 times in the litterature)
BrdU (Roche 11170376001) The antibody specifically binds to bromodeoxyuridine and crossreacts with iodouridine (10%). Anti-bromo-deoxyuridine does not crossreact with fluorodeoxy-uridine, nor with any endogenous cellular components such as thymidine or uridine. Anti-Bromodeoxyuridine shows 10% cross reaction with iodo-deoxyuridine, but no cross reaction to fluoro-deoxyuridine. No cross reaction to any endogenous thymidine or uridine. Cross reactivity with 5-Br-UTP has not been tested but it is suggested that there is a good chance for reaction, because the only difference is an absent hydroxyl group on the ribose distal to the bromine substitution. (cited 36 times in the litterature)
Cleaved Caspase3 (Cell signaling CST9661) Cleaved Caspase-3 (Asp175) Antibody detects endogenous levels of the large fragment (17/19 kDa) of activated caspase-3 resulting from cleavage adjacent to Asp175. This antibody does not recognize full length caspase-3 or other cleaved caspases. This antibody detects non-specific caspase substrates by western blot. (cited 9976 times in the litterature)
P-CREB S133 (Cell signaling CST9198) Phospho-CREB (Ser133) (87G3) Rabbit mAb detects endogenous levels of CREB only when phosphorylated at serine 133. The antibody also detects the phosphorylated form of the CREB-related protein, ATF-1. (cited 1158 times in the litterature)
CREB (Cell signaling CST9197) CREB (48H2) Rabbit mAb detects endogenous levels of total CREB-1 protein. The antibody does not cross-react with other ATF/CREB family members. Non-specific staining of components along the retinotectal pathway was observed by immunofluorescence in fixed frozen mouse tissue. (cited 922 times in the litterature)
CYP21 (Sigma Aldrich HPA 048979) Every Prestige Antibody is tested in the following ways:IHC tissue array of 44 normal human tissues and 20 of the most common cancer type tissues. Protein array of 364 human recombinant protein fragments. (cited twice in the litterature)
DAB2 (BD bioscience 610464) routinely tested in western blot, tested during development in immunofluorescence by BD bioscience. (cited 4 times in the litterature)
P-DRP1 S637 (Cell signaling CST4867) Phospho-DRP1 (Ser637) Antibody detects endogenous levels of DRP1 only when phosphorylated at Ser637. (cited 134 times in the litterature)
P-DRP1 S616 (Cell signaling CST3455) Phospho-DRP1 (Ser616) Antibody detects endogenous levels of DRP1 only when phosphorylated at Ser616. (cited 269 times in the litterature)
DRP1 (NOVUS NB55237) reviewed thrice with 5/5 stars. (cited 36 times in the litterature)
GAPDH (NOVUS NB300221) reviewed 19 times with 4.4/5 stars. (cited 361 times in the litterature)
GATA6 (Cell signaling CST5851) GATA-6 (D61E4) XP® Rabbit mAb recognizes endogenous levels of total GATA-6 protein. (cited 82 times in the litterature)
GFP (invitrogen A11122) This Antibody was verified by Relative expression to ensure that the antibody binds to the antigen stated (cited 1506 times in the litterature)
GFP (Abcam Ab5450) Pure GFP protein, or cells known to overexpress GFP used as positive control (cited 183 times in the litterature)
HA (Abcam ab9110) ELISA: The anti HA diluted 1:70.000 gave an O.D.=1.0 in a 15 minute reaction against peptide conjugated with a different carrier than used for anti peptide purification. HRP conjugated Goat anti rabbit IgG was used and TMB was the substrate. (cited 906 times in the litterature)
Ki67 (Abcam Ab15580) Knockout validated (cited 2869 times in the litterature)
Laminin (Sigma Aldrich L9393) Specificity of the anti-laminin antibodies is determined by indirect immunofluorescent labeling of formalin-fixed, paraffin-embedded human or animal tissue sections, and by dot blot immunoassay. By indirect immunofluorescence the antibody demonstrates specific basement membrane staining of enzymatically unmasked human and animal tissue. In the dot blot immunoassay the rabbit anti-laminin antibody reacts with laminin but not with fibronectin, vitronectin, collagen IV, or chondroitin sulfate types A, B, and C. The affinity isolated antibody to laminin will react with laminin of human, mammal, avian, reptilian, and amphibian sources. (cited 1487 times in the litterature)
NR2F2 (Perseus PPDH7147D00!)
PKA Cαβ (BD bioscience 610981) routinely tested in western blot, tested during development in IHC by BD bioscience. (cited 5 times in the litterature)
SF1 (Cosmo Bio KAL-KO611) knock-out validated doi:10.1242/dev.087247
SUMO1 (Cell signaling CST4930) SUMO-1 Antibody detects recombinant SUMO-1 and endogenous levels of sumoylated proteins (e.g. SUMO-1-RanGAP, 90kD). (cited 77 times in the litterature)

SUMO2/3 (8A2) validated against other SUMO2/3 antibodies https://doi.org/10.1101/2022.03.19.484974
SUMO2/3 (Abcam Ab3742) Recognises 2 bands representing Sumo 2 and Sumo 3 at 15 and 18kDa in Hela Nuclear extract by Western blotting.(cited 83 times in the litterature)
TH (Antibodies online AA 30 100) Specific for TH according to antibodies online website
TH (Chemicon AB 152) POSITIVE CONTROL: Brain (corpus striatum, sympathetic nerve terminals) and adrenal glands. NEGATIVE CONTROL: Liver.
Tomato/RFP (Rockland 600-401-379) This product was prepared from monospecific antiserum by immunoaffinity chromatography using Red Fluorescent Protein (Discosoma) coupled to agarose beads followed by solid phase adsorption(s) to remove any unwanted reactivities. Expect reactivity against RFP and its variants: mCherry, tdTomato, mBanana, mOrange, mPlum, mOrange and mStrawberry. Assay by immunoelectrophoresis resulted in a single precipitin arc against anti-Rabbit Serum and purified and partially purified Red Fluorescent Protein (Discosoma). No reaction was observed against Human, Mouse or Rat serum proteins. (cited 924 times in the litterature)
TRIM28 Bethyl (BETA700-014-T)
P-TRIM28 S473 (Biolegend BLE654101) WB - Quality tested, ICC - Verified

Eukaryotic cell lines

Policy information about cell lines and Sex and Gender in Research

Cell line source(s)	ATC7 adrenocortical cell line developed in our lab and characterised by Ragazzon et al. Endocrinology 2006 (PMID: 16439455). The original source is thus available at the institute GReD CNRS INSERM Université Clermont Auvergne, Clermont-Fd, France
Authentication	ACTH responsiveness of glucocorticoids released in the medium: ATC7 cells were cultured in presence of 10-8M ACTH for 24h, media were collected and corticosterone production was measured by ELISA. Forskolin (10-5M, 6h treatment) responsiveness of Mc2r, Scarb1, Star, Akr1b7 and Cyp11b1 gene expression was tested by RT-qPCR.
Mycoplasma contamination	tested negative for Mycoplasma
Commonly misidentified lines (See ICLAC register)	No commonly misindentified lines was used.

Animals and other research organisms

Policy information about studies involving animals; ARRIVE guidelines recommended for reporting animal research, and Sex and Gender in Research

Laboratory animals	Wild-type or genetically modified mice (Mus musculus) were used. All mice were on a mixed sv129-C57BL/6 genetic background. All experiments were done with male and female mice. All controls were littermates. Informationabout sex and animal numbers can be found in the figure, figure legends and methods. Age was 3-40 weeks of age. Genetically modified mouse lines used were: B6.129(Cg)-Gt(ROSA)26Sortm4(ACTB-tdTomato,-EGFP)Luo/J named R26RmTmG in the manuscript; Sf-1/Cre (High) Bingham et al. 2006 (PMID: 16937416) named Sf1 (Nr5a1)-Cre in the manuscript; Senp2fx/fx Qi et al. 2014 (PMID: 25189211) named Senp2fl/fl in the manuscript; AS+/Cre knockin Freedman et al. 2013 (PMID: 24035414); Prkar1atm1.2Lsk/J Kirschner et al. 2005 (PMID: 15930266) named Prkar1fl/fl in the manuscript.<br>Senp2cKO mice genotype is Senp2fl/fl::R26RmTmG/mTmG::Sf1-Cre; Prkar1acKO mice genotype is Prkar1afl/fl::Sf1-Cre; Senp2,Prkar1adcKO mice genotype is Senp2fl/fl::Prkar1afl/fl::R26RmTmG/mTmG::Sf1-Cre
Wild animals	no wild animals was used.
Reporting on sex	Most experiments were done in both sexes and reported separately
Field-collected samples	No field-collected samples was collected.
Ethics oversight	Mouse experiments were conducted according to French and European directives for the use and care of animals for research purposes and were approved by the Comité d'Éthique pour l'Expérimentation Animale en Auvergne (project agreement #211522019061912052883), C2EA-02, at Institut National de Recherche pour l'Agriculture, l'Alimentation et l'Environnement, Research Centre Clermont-Theix, France (C2E2A).

Note that full information on the approval of the study protocol must also be provided in the manuscript.
